# The Hierarchy of Walking Needs and the COVID-19 Pandemic

**DOI:** 10.3390/ijerph18147461

**Published:** 2021-07-13

**Authors:** Mohammad Paydar, Asal Kamani Fard

**Affiliations:** 1 Escuela de Arquitectura Temuco, Facultad de Humanidades, Universidad Mayor, Av. Alemania 281, Temuco 4780000, Chile; 2 Universidad Católica del Maule, San Miguel 3605, Talca 3460000, Chile; asal.kamanifard@gmail.com

**Keywords:** COVID-19, resilience, walking behaviour, hierarchy of walking needs, built environment

## Abstract

More than 150 cities around the world have expanded emergency cycling and walking infrastructure to increase their resilience in the face of the COVID 19 pandemic. This tendency toward walking has led it to becoming the predominant daily mode of transport that also contributes to significant changes in the relationships between the hierarchy of walking needs and walking behaviour. These changes need to be addressed in order to increase the resilience of walking environments in the face of such a pandemic. This study was designed as a theoretical and empirical literature review seeking to improve the walking behaviour in relation to the hierarchy of walking needs within the current context of COVID-19. Accordingly, the interrelationship between the main aspects relating to walking-in the context of the pandemic- and the different levels in the hierarchy of walking needs were discussed. Results are presented in five sections of “density, crowding and stress during walking”, “sense of comfort/discomfort and stress in regard to crowded spaces during walking experiences”, “crowded spaces as insecure public spaces and the contribution of the type of urban configuration”, “role of motivational/restorative factors during walking trips to reduce the overload of stress and improve mental health”, and “urban design interventions on arrangement of visual sequences during walking”.

## 1. Introduction

Resilience refers to a system’s ability to efficiently absorb shocks [1]. The recent pandemic of COVID-19 has influenced many aspects of our daily life. It has meant various changes to the physical and social arrangements of our cities in order to increase urban resilience in the face of this pandemic. In regard to urban transport, in many cities, the provision of public space and infrastructure for the development of active travel-including walking and cycling-has been adopted as the main approach to increase urban resilience in the face of this pandemic [1]. These active transport modes, especially walking, are the most sustainable modes of transport. More than 150 cities have expanded emergency cycling and walking infrastructures as of late April 2020 [2]. Many cities such as Auckland, Barcelona, Bogota, New York, Quito and Rome, have been aiming to improve city infrastructure to facilitate socially distanced walking and cycling and other cities such as Montreal, Oakland, Portland, San Diego, San Francisco and Vienna are trying to create slow/safe street networks that prioritize pedestrians and cyclists and limit car access [2]. For instance, re-timing traffic lights was adopted in Brussels to give more time for pedestrians and cyclists and avoid crowding at junctions [2].

Although, as of mid-2021, it is yet unclear the duration of this pandemic, it seems that this approach toward active travel, and especially walking, is, from a long-term point of view, due to the high expense invested in this area in many cities. Walking is the cheapest and most sustainable mode of transport. This tendency of policy makers as well as inhabitants toward walking, has led to it becoming the predominant mode of transport in daily trips for many people. In regard to the relationship between the walking behaviour of citizens and their walking needs, there are five levels of needs that are considered within the walking decision-making process. These needs progress from the most basic need, feasibility (related to personal limitations), to higher-order needs (related to urban landscape) that include accessibility, safety, comfort, and pleasure, respectively [3]. Within this hierarchical structure, an individual would not typically consider a higher-order need in his or her decision to walk if a more basic need was not already satisfied [3].

Due to the situation imposed by COVID-19, since people may choose more often to walk rather than use other modes of transport, the more basic needs of walking such as accessibility and safety may play a more substantial role in the decision to walk when compared with the situation before the pandemic. Consequently, the relationship between choosing to walk and the hierarchy of walking needs may also be changed substantially. Moreover, in addition to choosing to walk and walking behaviour, walkability is the other relevant term in regard to walking patterns in the relevant studies. The walkability of a neighbourhood generally refers to the extent to which that neighbourhood provides adequate conditions for walking [4] and previous studies have used different compositions of built environmental attributes to measure the walkability index [5,6,7,8,9]. The current situation with regard to COVID-19, meaning that more basic walking needs may play a more important role in the daily walking patterns of inhabitants, may also lead to the need to re-examine and re-define the walkability of our neighbourhoods.

These changes in the relationship between the hierarchy of walking needs and walking behaviour, due to the situation imposed by COVID-19 need to be addressed. A recognition of these changes should lead to the improvement of walkability as well as walking behaviour during and after COVID-19 which will ultimately contribute to increase the resiliency of walking environments in the face of such a pandemic. The question raised in this regard is how to improve walking behaviour in relation to different levels in the hierarchy of walking needs regarding the current situation imposed by COVID-19. The next section tries to answer this question through considering the interrelationships between the different aspects concerning walking-imposed by this pandemic- and different levels in the hierarchy of walking needs.

## 2. The Interrelationships between Different Aspects Related to Walking in the Context of the Pandemic and Different Levels in the Hierarchy of Walking Needs

To answer the question of this study, there is a need to recognize the basic aspects of walking behaviour in the context of the pandemic. In this regard, the first aspect is the relationship between walking behaviour and density or overcrowding—as one of the main factors related to walking behaviour during this pandemic—in order to maintain physical distance and thus physical health. The second aspect is the role of crowded spaces in increasing stress during walking which in turn contributes towards mental health disorders of citizens. The third aspect is the role of walking environments in neutralizing and reducing the overload of stress generated by all the conditions imposed by this pandemic and which therefore contributes to enhancing the mental health of citizens as well.

**Figure 1:**
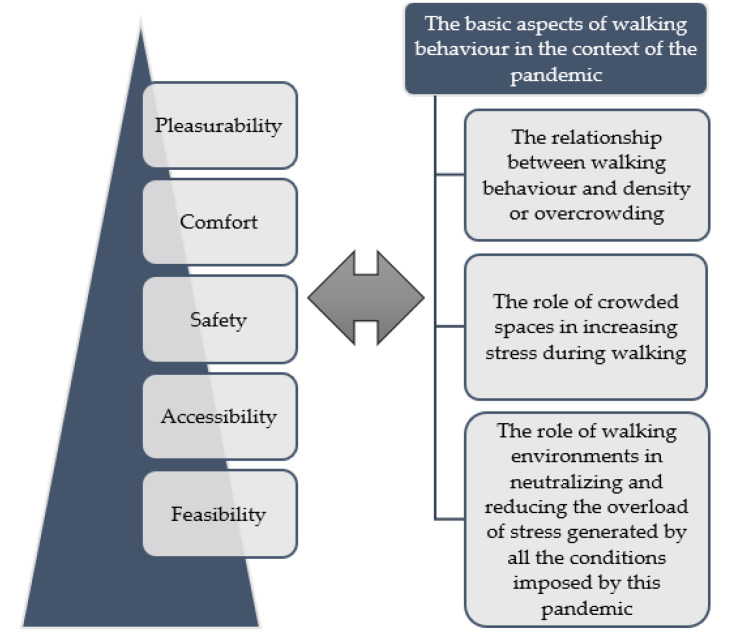
Interrelationship between different aspects of walking in the context of the pandemic and different levels of the hierarchy of walking needs.

The following subsections focus on these aspects and their interrelationships with different levels in the hierarchy of walking needs in order to address the new requirements for the improvement of walking behaviour in the context of the pandemic (Figure 1).

The first subsection focuses on the influence of density and crowding on the level of contagion and stress. This subsection considers the main concepts of psychological stress theory—including primary appraisal and coping—and their relationships with the main concepts raised from the studies on crowding.

The second subsection considers and discusses the interrelationships between comfort—as one of the levels in the hierarchy of walking needs—and the stress caused by crowded spaces. In this subsection, first, the main factors in regard to creating and increasing the sense of comfort during walking are introduced. Then, the changes related to the influence of these factors on the sense of comfort made by this pandemic—from the empirical and theoretical standpoints—are discussed.

The third subsection reviews a major debate in regard to the relationship between urban configuration and insecurity—as the other level in the hierarchy of walking needs—due to the similarity found between unsafe public places before this pandemic and crowded spaces during this pandemic. In this regard, two opposing concepts of designing public spaces in relation to the security and fear of crime are introduced and discussed. In addition, the contribution of urban land use patterns to the relationship between the type of urban configuration and level of the contagion as well as stress are considered. Additionally, the role of theory of urban fabrics in this process is discussed.

The next subsection reviews which environmental factors along the pathways contribute to the neutralization and reduction in stress caused by current conditions in the context of the pandemic. Accordingly, the role of pleasurability—as the last categorization of walking needs—and its contributing factors in reducing stress during walking is discussed. Then, special attention is given to the natural environments and urban greenery as the main motivational/restorative physical factors during walking. In this regard, the main theories, including “psycho-physiological stress reduction theory” and “Attention Restoration Theory”, considering the restorative impacts of greenery and natural environments during walking, are reviewed. In addition, the role of social-related factors—as a part of the motivational/restorative factors during walking—in reducing stress, especially for elderly people, is considered as well.

Finally, since walking trips include consecutive visual sequences, the interrelationships between visual as well as aesthetic factors along these pathways, as well as consecutive visual sequences, through relevant urban design theories including “urban picturesque theory” and “prospect-refuge theory” are discussed.

### 2.1. Density, Crowding and Stress during Walking

The creation of a compact city which maximizes the active use of space and land, is the main approach to creating and maintaining sustainable urban environments and factors including density, diversity and mixed land uses are key to this process [10,11,12]. Creating a compact city is reckoned to be one of the approaches for creating resilient cities as well [10]. However, high-density cities could be more vulnerable to the spread of infectious diseases as has been shown during the COVID-19 pandemic. Many people assume that contagion risk increases with density (the number of people per unit of land), but that is not necessarily true; the risk is actually associated with crowding (the number of people within a limited space) [1]. The widening of walkways was the main approach in regard to adapting infrastructure for walking during the recent pandemic and it was applied by many cities in order to decrease the real level of crowding in different parts of the pedestrian network.

**Figure 2:**
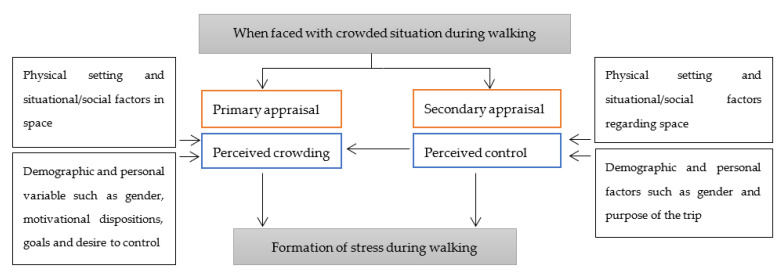
The process of stress formation when faced with crowded spaces during walking.

In addition to crowding and its influence on physical health, the stress created by crowded spaces during the pandemic is the main issue to arise from it. Stress as a relational concept is viewed as a relationship between individuals and their environment [13,14]. Stress is generated in a situation where a person evaluates something that is important for his/her wellbeing during his/her relationship with the surrounding environment and that situation exceeds their biological, psychological or social resources for coping with it [15,16]. Thus while faced with a new stress-generative situation, two basic forms of appraisal based on different sources of information are formed. Primary appraisal concerns whether something of relevance to the individual’s wellbeing occurs, whereas secondary appraisal concerns coping mechanisms [13]. Indeed, these are two components which contribute to the formation of stress while faced with stress-generative situations like crowded spaces at the time of this pandemic. The effects of these two components on the formation of stress while faced with crowded spaces, is supported by the results of studies on crowding as well [17,18]. Perceived crowding or one’s perception of crowding has an impact on how people perceive a crowded situation which could be different or similar to the real level of crowding. In fact, for a fixed level of crowding, perceived crowding can vary depending on the situations and the people involved. Furthermore, people generally feel better and have better mental health when they can control their surroundings [19,20,21]. Perceived control is the perception people have about how they can control and maintain their wellbeing when faced with crowded spaces. In this regard, as was shown in Figure 2, perceived control has been introduced as a crucial intervening variable between actual physical crowding and perceived crowding [17,18]. In fact, perceived control is the mediator in the relationship between real crowding and perceived crowding (Figure 2). Additionally, it has an important role in the formation of stress of pedestrians. Indeed, perceived crowding corresponds to primary appraisal and perceived control corresponds to secondary appraisal when faced with crowded spaces (Figure 2).

In addition to the physical setting of crowding in crowded spaces, demographic and personal variables such as gender, goal, motivational dispositions and desire for control, also contribute to both perceived crowding and perceived control when faced with crowded situations [13,22,23,24] (Figure 2). For instance, the trip purpose whether utilitarian or hedonic, in nature, moderates the effect of perceived crowding on satisfaction in a restaurant [24]. Likewise, previous studies on walking behaviour have also demonstrated the role of purpose of walking—whether walking to reach a destination or walking for recreation—in associations between environmental factors and levels of walking [25,26,27]. Thus, it can be inferred that the purpose of walking trips—whether utilitarian or recreational walking trips—may also influence the associations among crowded spaces, perceived crowding and the formation of stress during walking.

### 2.2. Sense of Comfort/Discomfort and Stress in Regard to Crowded Spaces during Walking Experiences

Comfort as the fourth level of needs for walking, refers to a person’s level of ease, convenience, and contentment [3]. The sense of comfort during walking may be affected by environmental qualities that either facilitate walking or remove factors that might make the walk stressful [3]. Sense of comfort/discomfort has become one of the most relevant perceptual factors influencing the level of stress while encountering crowded spaces during walking in this pandemic. Before this pandemic, the expected number of pedestrians in crowded spaces contributed to an increase in stress only in conditions where the number of interactions with other pedestrians effectively impeded pedestrian movement in such areas [28]. Indeed, an average number of expected interactions with other pedestrians provides more opportunities for reinforcing social interaction and the vitality of urban public spaces and therefore, it was recommended by studies in urban design before this pandemic [29]. For instance, the fundamental principles of the New Urbanist movement concern what are positioned as “liveable” spaces at a human scale. Then, walking is a practice that encourages “social mixing”, “community cohesion”, and “social interaction”; and thereby may create more livable public spaces [30].

**Figure 3:**
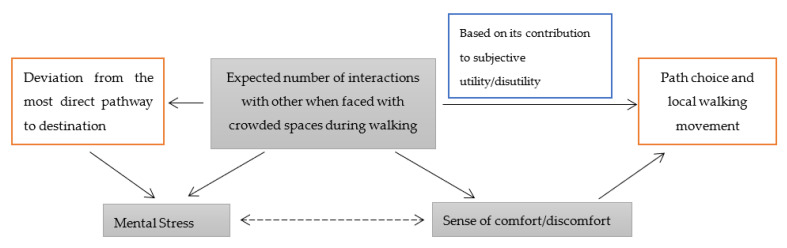
The process of intervention of sense of comfort/discomfort to stress and walking movement while encountering with crowded spaces during walking in this pandemic.

The situation has changed dramatically due to this pandemic in which crowding and the expected number of interactions with others have become the main source of stress during walking. The expected number of interactions with other pedestrians has always been known to be one of the main factors in determining the level of comfort/discomfort along pathways [3,28,31]. Comparing these relationships during this pandemic shows that when the feeling of comfort is reduced—due to the presence of other pedestrians in crowded spaces—such a feeling quickly turns into stress. Such a relationship did not exist before this pandemic. This shows that a high convergence has been created between the factors including the expected number of interactions in crowded spaces, sense of comfort/discomfort and level of stress when faced with crowded spaces during this pandemic (Figure 3).

In addition, according to pedestrian behavioural modeling, environmental factors, depending on their role in subjective utility/disutility of path choice as well as choice of walking patterns of pedestrians in local environments, contribute to their level of walking [32,33,34]. Several of these physical factors along the path which contribute to subjective utility/disutility, influence the sense of comfort/discomfort of pedestrians as well. Examples of these physical factors are the presence of obstacles during walking and the expected number of interactions with other pedestrians which contribute to both subjective utility/disutility as well as pedestrians’ sense of comfort/discomfort during their walking [28]. Thus, the expected number of interactions with other pedestrians contributes to both utility/disutility as well as pedestrians’ sense of comfort/discomfort (Figure 3). Consequently, sense of comfort could function as a mediator in the relationship between expected number of pedestrians and their path choice as well as their local walking patterns (Figure 3). Such interrelationships were emphasized in previous studies related to walking as well [35].

Furthermore, empirical studies on the micro scale walking behaviour of pedestrians, before this pandemic, have shown that people prefer to maintain a distance of around 0.8 to 0.9 m between themselves and other pedestrians as a comfortable walking distance from others during their walking [36,37]. The findings of these studies have been applied in predicting walking movement through micro scale modeling of pedestrian movement [37,38]. This comfortable physical distance from other pedestrians has changed dramatically due to this pandemic. However, new empirical studies are required to understand the actual adopted physical distance as well as the comfortable physical distance from other pedestrians in order to be used in modeling and predicting the micro scale walking behaviour of pedestrians in each context.

Finally, Osaragi [39] found that there are two sources of discomfort and stress including the presence of other pedestrians—as stated before—and deviation from the shortest path to the destination in a micro scale environment. For instance, the former source of stress is generated in a crowded metro station where the pedestrian cannot walk along the shortest path to the destination (e.g., ticket booth). Thus, a higher number of interactions with others in crowded spaces may also influence deviation from the shortest path to the destination as the other source of stress in such crowded spaces (Figure 3). In this pandemic it is likely that high percentages of walking trips are made in order to reach a destination. Pedestrian behavioral models that focused on an activity-based approach also emphasized the importance of places which generate activities during walking trips [34]. These places are indeed the medial or final destinations during walking trips. In this regard, the impact of deviation from shortest path to the destination on generation of stress should also be considered in future empirical and theoretical studies which consider the impact of such micro scale crowded spaces on stress during walking.

### 2.3. Crowded Spaces as Insecure Public Spaces and the Contribution of the Type of Urban Configuration

During this pandemic, crowded pedestrian spaces have become unsafe public places which may contribute to a higher contagion of COVID-19 as well as greater stress among residents. In this regard the contribution of these crowded spaces towards higher levels of contagion—as well as increased stress—is similar to that of insecure public places before this pandemic in increasing the level of insecurity, fear of crime and stress. Security refers to whether a person feels safe from the threat of crime [3]. It is the third level of needs for walking [3]. Previous studies have demonstrated the influence of insecurity and fear of crime on walking behaviour [40,41]. Several studies have found that effective measures of fear of crime are associated with poorer mental and physical health, although effect sizes are generally modest [42,43,44,45,46]. Similarly, theories regarding the determinants of mental health have introduced crime as a major explanatory factor.

In addition, previous studies on crime and fear of crime in the urban environment have tried to clarify the relationship between the level of crime and fear of crime with urban texture or urban configuration [47,48]. The relationship between the type of urban configuration and the level of contagion as well as its contribution towards stress needs to be addressed in this pandemic as well. In this regard, the following debate needs to be reviewed. One of the major current debates on the relationships between environmental factors and crime as well as fear of crime, has to do with two opposing concepts of designing public spaces including permeable space versus defensible space [47]. A number of studies have reported that environmental factors that promote permeable neighbourhood settings, such as better street connectivity and mixed land uses improve security through encouraging people to be present on the streets and providing more informal public surveillance [49,50,51,52]. Other studies suggest that increased permeability and mixed land uses are associated with increased crime [53,54,55,56] and that homogenous neighbourhoods with restricted vehicular and pedestrian access are safer [57,58]. This is supported by criminologists who claim that mixed uses and high residential density generates more contact between potential offenders and potential victims; thus increasing the rate of crime [48,59,60]. Therefore, there is a debate regarding the role of the type of urban setting, whether permeable or defensible, on increasing the crime rate as well as fear of crime.

**Figure 4:**
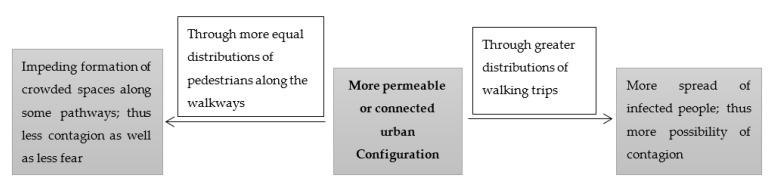
The process of the role of type of urban configuration on the level of contagion as well as stress.

In regard to this pandemic, on the one hand, more permeable urban settings through a greater distribution of walking trips may contribute to a greater spread of infected people and thus a higher possibility of contagion; and on the other hand, more connected or permeable urban settings through more equal distributions of pedestrians along different walkways may impede the formation of crowded spaces along the pathways, therefore contributing to less contagion and less fear which also then contributes towards a reduction in stress (Figure 4). This is similar to the existing debate, previously mentioned, on the relationship between the type of urban setting and the level of crime. Thus far, from the few studies on this topic, the contribution of connected urban spaces towards the increased spread of this pandemic has been demonstrated [61]. However, more studies are required regarding the relationship between the type of urban configuration and the level of contagion as well as stress in regard to walking behaviour.

Furthermore, in addition to the role of the type of urban configuration on the distribution of pedestrians along different walkways, the pattern of land uses as attractors of pedestrians—in terms of their numbers and distributions—influences the distribution pattern of pedestrians in each area. Thus, a combination of type of urban configuration and urban land uses—as attractors of pedestrians—determines the distribution pattern of pedestrians in each urban area. These characteristics may be differentiated among different urban sectors. For instance, most of the city centers include land uses such as city administration offices many of which can be counted as attractors of pedestrians. In such crowded urban areas, the permeable urban configuration works better than the defensible type of urban configuration resulting in less spread of the pandemic. This is because in such crowded urban areas, more permeability leads to a more equal distribution of pedestrians and less formation of micro scale crowded spaces along different walkways which contribute to less spread of the pandemic as well as less stress.

Therefore, the relationship between the type of urban configuration and level of contagion as well as stress could be differentiated based on different types of urban configuration as well as different patterns of land uses in each urban sector. Among the existing theories, the theory of urban fabrics supports the need to study different urban sectors based on their dominant urban form. This theory suggests that different planning approaches are needed in different parts of the city based on their dominant urban form [62]. Further studies are required to understand how to organize the movement of pedestrians in different urban configurations in order to increase the resiliency of walking environments in the face of this pandemic as well as a similar crisis situation in the future.

### 2.4. The Role of Motivational/Restorative Factors during Walking Trips to Reduce the Overload of Stress and Improve Mental Health

Previous studies have mentioned the need for more convergence between public health and urban planning including the planning of walking environments to improve public health among urban populations [63]. This pandemic reminds us of such a need more than ever. In addition to the role of crowded spaces in increasing contagion, stress increased dramatically during this pandemic due to fear of such crowded spaces during walking and all the other limitations imposed by COVID-19. Thus, we have been faced with an overload of stress during this pandemic.

**Figure 5:**
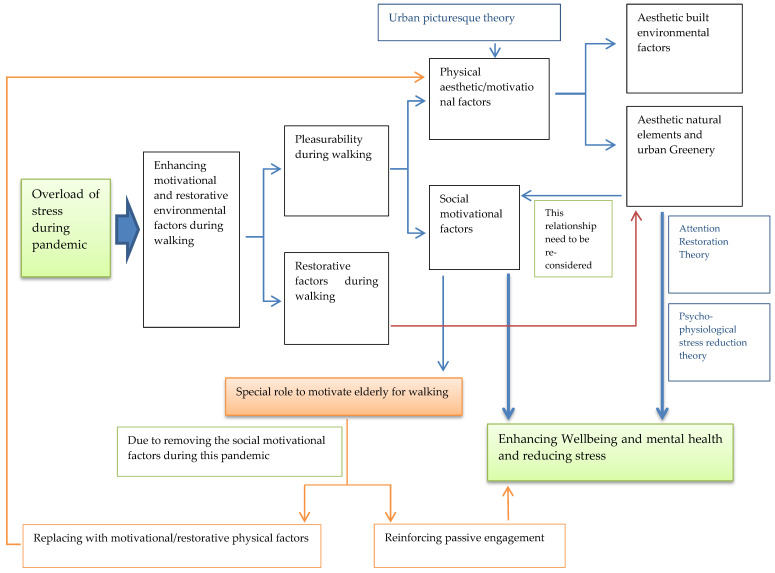
The process of the role of motivational/restorative environmental factors on neutralizing and reducing stress overload.

Walking itself can decrease symptoms of depression and anxiety as well as lead to improvements in individual quality of life [64]. However, in addition to walking, one of the main roles of environmental factors during this pandemic is to reduce such an overload of stress in order to improve the public health of citizens. Studies on walking behavior have demonstrated the contribution of motivational factors to walking behaviour. Most of these factors are those related to pleasurability during walking (Figure 5). Pleasurability as the last categorization of walking needs refers to the level of appeal that a setting provides with respect to a person’s walking experience [3]. It is also related to how enjoyable and interesting an area is for walking [3]. Several physical related factors such as vegetation and social related factors such as outdoor dining areas have been demonstrated as relating to pleasurability which contribute to greater preference for walking as well as improvement of walking behaviour [25,31,35,65,66,67,68,69]. According to urban picturesque theory, the majority of these features and factors can be counted as aesthetic natural and built environment factors as well [70] (Figure 5).

Among the motivational/restorative physical factors during walking, natural environments as well as urban greenery have a remarkable impact on reducing stress and improving mental health [19,31,71,72,73,74,75] (Figure 5). However, restorative experiences in waterside environments and extensively managed natural settings (mainly urban woodlands) are stronger compared to favorite places in built urban settings or green spaces of urban settings (mostly parks) [76]. “Psycho-physiological stress reduction theory” and “Attention Restoration Theory” provide the theoretical basis for such a restorative effect of interaction with natural environment as well as urban greenery [77] (Figure 5). “Psycho-physiological stress reduction theory” proposes that contact with nature (e.g., views of natural settings) can have a positive effect for those with high levels of stress, by shifting them to a more positive emotional state [78,79]. “Attention Restoration Theory” suggests that involuntary attention given to interesting and rich stimuli in natural settings helps to improve performance in cognitively demanding tasks [80,81]. In addition, experimental and empirical studies have found evidence regarding the association between restoration from stress as well as mental fatigue and exposure to natural environments and urban greenery [71,72,74,75,82,83,84,85].

Furthermore, motivational/restorative factors during walking include social-related factors as well (Figure 5). There is the well-known protective effect of social relationships on health and wellbeing, while social isolation is a known predictor of mortality [79,86,87,88] (Figure 5). These social related factors play an important role in motivating pedestrians, especially elderly people to walk (Figure 5). The social environment refers to the influence that friends and family can have on an individual’s walking [89]. According to Krogstad, Hjorthol and Tennøy [90], the “want to” dimension of walking—including the motivational aspect of walking—makes the walking enjoyable for elderly people and it is one of the most important dimensions to improve their walking. The social related factors—as part of the motivational factors, especially for walking of elderly—include companionship of family or friends for walking [90,91,92], encouragement to walk by family and friends [93,94], neighborhood social cohesion [89,95]; watching people or the viewing of others’ activities [90] and community engagement including development of interpersonal relationships (social networks) as well as meeting others during walking and social support from neighbors [95,96,97]. Supportive social networks may also decrease the perceived impact of physical barriers to engage in physical activity such as walking [98]. Furthermore, the social dimension of public spaces is one of the main components that contribute to generating and enhancing the place attachment and sense of place as well [99,100].

Elderly people are the most vulnerable group in regard to COVID-19 and fear of being infected by the virus together with staying at home alone may seriously increase stress among the elderly. Therefore, related social factors are of special importance for the improvement of mental health in elderly people during this pandemic. According to Maslow [101] these social interrelationships are indeed the third level of human needs as well. In this context, we have seen how the pandemic has affected and destroyed those social relations. Therefore, what is the solution to reviving the role of these social motivational factors at the time of the pandemic to improve mental health as well as walking? One solution is to strengthen the possibility of passive engagement rather than active engagement with the environment (Figure 5). Passive engagement with the environment involves the need for connecting with the surroundings without being actively involved [102]. The most common passive activity is “people watching”. According to Gehl [103], the opportunities to see, hear, and talk are a prerequisite for communication between people in the city spaces. In this regard, the design of public spaces should be carried out in a way that allows for the viewing of other people without the possibility of direct contact between pedestrians. The other solution is to reinforce the motivational/restorative physical factors instead of social motivational factors during walking (Figure 5). 

Finally, one of the ways in which green space contributes to wellbeing and mental health is through its influence on increasing social interactions in the neighborhood (Figure 5). Green space can play an important role in the improvement of perceived social cohesion and fostering social interactions which also help to promote a sense of community [104,105,106]. As stated before, reinforcing these social factors contributes to improving mental health as well [88]. However, due to the gradual elimination of social interactions—as a result of this pandemic—the socially related health impact of green spaces has been removed [107]. Therefore, in the current situation, the use of green spaces for their impact on mental health through enhancement of social interaction needs to be re-considered (Figure 5).

### 2.5. Urban Design Interventions on Arrangement of Visual Sequences during Walking

Walking is a movement and it is different from stationary activities such as sitting. Therefore, the effect of motivational/restorative factors on health as well as walking behaviour depends on the effect of compositions of these factors within consecutive visual sequences during walking. Urban design theories have addressed this issue.

According to urban picturesque theory, some compositions of spatial form and specific design features invoke a more intense aesthetic experience than other compositions which contribute to increasing pedestrian activity and walking trips [31,70]. Based on this theory, the balance between full visual connection between the different visual sequences along the path and semi-hidden views among these visual sequences is one of the core characteristics of urban aesthetic visual sequences which contributes to more intense aesthetic experiences. The former type contributes to increasing unity, clarity and controllability and the second type encourages pedestrians to explore new things along the consecutive visual sequences during walking.

The other theory in regard to the arrangement of visual sequences during walking is prospect-refuge theory. This theory points out that an environment which affords a certain amount of prospect (open view) and refuge (concealment, protection) offers an evolutionary advantage to humans [108]. However, previous studies found that such a balance between open view and concealment is commonly applicable in natural environments [109]. In addition, urban studies supported the significance of prospect or open view, and were more neutral about refuge or concealment [109,110,111]. For instance, urban areas characterized by large amounts of refuge (concealment) and minimal prospect, tend to evoke the highest degree of fear amongst individuals as well as having the highest proportion of crime [112,113]. Considering the situation imposed by COVID-19, there is a need to reduce concealment and increase visual connectivity among the continuous visual sequences during walking. This is in order to enhance controllability as well as the perceived control of pedestrians during their walking trips which, as previously stated, are the main factors for reducing contagion as well as the stress of pedestrians during this pandemic.

## 3. Conclusions

Through the current situation imposed by this pandemic, the relationship between patterns of walking behaviour with different levels in the hierarchy of walking has changed substantially. These changes need to be addressed. Recognition of these changes leads to the improvement of walkability as well as walking behaviour both during and outside of COVID-19, which will finally contribute to more resilient cities in terms of walking behaviour in the face of such a pandemic. This study was designed as a theoretical and empirical literature review seeking to improve the walking behaviour in relation to the hierarchy of walking needs within the current context of COVID-19. In this regard, three aspects were recognized as the basic aspects relevant to walking behaviour imposed by this pandemic. Next, the interrelationships between these aspects and different levels in the hierarchy of walking needs were considered and discussed in order to address the new requirements for improvements in walking behaviour in the situations imposed by this pandemic.

In regard to density as well as crowding, it was explained that contagion risk increases with crowding and not people density in public spaces. Moreover, respecting the impact of crowded spaces on the formation of stress, the two primary factors of perceived crowding and perceived control were introduced. Furthermore, the purpose of the walking trips—whether for transport or recreation—was also introduced as a factor that should be considered by future relevant studies.

Regarding the impact of sense of comfort—as one of the levels in the hierarchy of walking needs—upon stress and walking behaviour, it was shown that there is a high convergence between the factors: expected number of interactions in crowded spaces, sense of comfort/discomfort and level of stress when faced with crowded spaces during this pandemic. Furthermore, due to the notable changes in comfortable physical distance due to this pandemic, new empirical studies are required in each context to understand the actual adopted physical distance as well as the comfortable physical distance of pedestrians. It was also found that the deviation from the shortest path to the destination has been previously presented as a source of generation of discomfort as well as stress in micro scale walking environments. Thus, the role of deviation from the shortest path to destination on the generation of stress should also be considered in future empirical studies on walking in micro scale crowded spaces.

In regard to the relationship between crowded spaces and insecurity—as the other level in the hierarchy of walking needs—it was stated that the role of these crowded spaces in increasing contagion as well as stress is similar to the role of insecure public places before this pandemic in increasing the level of insecurity, fear of crime and stress. Previous studies on crime and fear of crime in the urban environment have tried to clarify the relationship between the level of crime and fear of crime with urban configuration. The relationship between urban configuration and the level of contagion as well as stress also needs to be addressed in light of the pandemic. In this regard a major debate on the relationship between type of urban configuration and crime rate was reviewed. This debate is on the impact of type of urban configuration whether permeable or defensible on level of crime as well as fear of crime. It was shown that a similar debate exists on the relationship between the type of urban configuration and the level of contagion as well as stress in the situations imposed by this pandemic. Further studies are required to clarify which type of urban configuration would contribute to less contagion and less stress in different urban sectors of each city.

Furthermore, motivational/restorative factors during walking may play a significant role in neutralizing and reducing the stress overload of inhabitants and enhancing their mental health during this pandemic. Most of the motivational/restorative factors during walking are those related to pleasurability—as one of the levels in the hierarchy of walking needs—which includes physical and social motivational factors during walking. Among the motivational/restorative physical factors during walking, natural environments as well as urban greenery have a notable impact on reducing stress and improving mental health. In this regard, the relevant theories were reviewed which support the restorative and mental health impact of natural elements and greenery on pedestrians during walking. Furthermore, the motivational/restorative factors during walking also include social factors and there is a well-known protective effect of social relationships on health and wellbeing, while social isolation is a known predictor of mortality. Such social factors are of particular importance for the mental health of inhabitants especially older people during this pandemic. Additionally, elderly people are the most vulnerable group in regard to COVID-19. However, the pandemic has adversely affected these social relations. In this regard the solutions to reviving the role of these social motivational factors at the time of the pandemic are to strengthen the possibility of passive engagement rather than active engagement with the environment and/or to reinforce the presence of motivational/restorative physical factors in walking environments instead of motivational social factors.

Finally, it was stated that walking is a movement and is different from stationary activities. Therefore, the effect of motivational/restorative factors on health as well as patterns of walking behaviour depends on the effect of compositions of these factors within consecutive visual sequences during walking. In this regard, the respected theories in urban design were reviewed. Additionally, it was shown that considering the situation imposed by COVID-19, it is more necessary to reduce concealment and increase visual connectivity among the continuous visual sequences during walking. This is in order to increase controllability as well as perceived control of pedestrians during their walking trips, which is the important factor during walking at the time of this pandemic.
